# New findings of the butterfly *Phengaris teleius* at the border between Hungary and Serbia (Lepidoptera: Lycaenidae)

**DOI:** 10.3897/BDJ.4.e8078

**Published:** 2016-03-09

**Authors:** Miloš Popović, Martina Šašić

**Affiliations:** ‡ HabiProt, Bulevar oslobođenja 106/34, 11040 Belgrade, Serbia; § University of Kragujevac, Faculty of Science, Department of Biology and Ecology, Radoja Domanovića 12, 34000 Kragujevac, Serbia; | Croatian Natural History Museum, Demetrova 1, 10000 Zagreb, Croatia

**Keywords:** Nature conservation, species distribution, Scarce Large Blue butterfly, Natura 2000

## Abstract

**Background:**

Due to its interesting life cycle, vulnerability and conservation importance, the butterfly *Phengaris teleius* is one of the most studied insects in Europe. It was discovered in Serbia only in 2012 and there were no data on its distribution from the south of Hungary.

**New information:**

*Phengaris teleius* was recorded for the first time in four localities in Hungary and in additional locality in Serbia. This suggests that the local populations are more interconnected than previousely thought and that conservation efforts should be done in collaboration between the two countries. All localities are inside protected areas and management measures for preserving several target species already exist. This represents a solid base for the conservation of newly discovered populations of *P. teleius*.

## Introduction

The Scarce Large Blue, *Phengaris teleius* (Bergsträsser, 1779), is distributed from Central Europe to Asia. The European part of its range is well known as the species received great attention over the last few decades due to its extraordinary myrmecophilous life cycle making it sensitive to habitat changes and therefore in risk of extinction (Settele et al. 2005, Wynhoff 1998). The species is now assessed as vulnerable in Europe (Van Swaay et al. 2010) and listed in Annex II and IV of the Habitats Directive (European Commission, No. 92/43/EEC). European Union member states have the obligation to monitor the status of this species, the size of its populations and its distribution range. Hungary has already designated Natura 2000 sites for the conservation of *P. teleius*, but none in the south-eastern part of the country, where the presence of the butterfly has not been recorded yet (Haraszty 2014). Serbia, as a candidate country, has an obligation to study the distribution and population trend of target species and to propose SCI areas (Sites of Community Importance) for its upcoming Natura 2000 network. *P. teleius* was discovered in 2012 in the very north of the country, some 50 km from the nearest known populations in Hungary (Popović et al. 2014). This triggered a more detailed study on the distribution of this species around the border between Hungary and Serbia and the results are summarised here.

## Materials and methods

Potential habitats of *Phengaris teleius*, containing the food plant of the butterfly (*Sanguisorba officinalis* L.) were identified using Google Earth satellite images and with the help of local rangers. In order to check whether *P. teleius* lives on the Hungarian side of the border, we visited four localities inside of the National Park Kiskunság on 6th of August 2015: 1. Rívó-erdő és semlyék, 2. Ásotthalmi láprét (Csodarét), 3. Csipak semlyék and 4. Domaszéki canal (north of Zákányszék). The distribution of *P. teleius* in Serbia has been studied since 2012 and here the results are summarised, wtih the addition of the recently discovered location.

## Taxon treatments

### Phengaris teleius

(Bergsträsser, 1779)

http://www.gbif.org/species/4535172

http://eol.org/pages/267710

http://www.faunaeur.org/full_results.php?id=441077

http://alciphron.habiprot.org.rs/pregled.php?grupa=8&vrsta=441077

#### Materials

- **Type status:** Other material. **Occurrence:** recordedBy: Miloš Popović; lifeStage: Adult; **Taxon:** kingdom: Animalia; phylum: Arthropoda; class: Insecta; order: Lepidoptera; family: Lycaenidae; genus: Phengaris; specificEpithet: teleius; scientificNameAuthorship: (Bergsträsser, 1779); **Location:** continent: Europe; country: Serbia; stateProvince: Vojvodina; decimalLatitude: 46.160419; decimalLongitude: 19.733821; geodeticDatum: WGS84; **Identification:** identifiedBy: Miloš Popović; **Event:** samplingProtocol: Observation; eventDate: 2013/08; year: 2013; month: 8; **Record Level:** type: Dataset; modified: WedWed/FebFeb/20162016; language: English; rightsHolder: Miloš Popović
- **Type status:** Other material. **Occurrence:** recordedBy: Miloš Popović; lifeStage: Adult; **Taxon:** kingdom: Animalia; phylum: Arthropoda; class: Insecta; order: Lepidoptera; family: Lycaenidae; genus: Phengaris; specificEpithet: teleius; scientificNameAuthorship: (Bergsträsser, 1779); **Location:** continent: Europe; country: Serbia; stateProvince: Vojvodina; decimalLatitude: 46.157306; decimalLongitude: 19.737926; geodeticDatum: WGS84; **Identification:** identifiedBy: Miloš Popović; **Event:** samplingProtocol: Observation; eventDate: 2013/08; year: 2013; month: 8; **Record Level:** type: Dataset; modified: WedWed/FebFeb/20162016; language: English; rightsHolder: Miloš Popović
- **Type status:** Other material. **Occurrence:** recordedBy: Miloš Popović; lifeStage: Adult; **Taxon:** kingdom: Animalia; phylum: Arthropoda; class: Insecta; order: Lepidoptera; family: Lycaenidae; genus: Phengaris; specificEpithet: teleius; scientificNameAuthorship: (Bergsträsser, 1779); **Location:** continent: Europe; country: Serbia; stateProvince: Vojvodina; decimalLatitude: 46.153171; decimalLongitude: 19.742282; geodeticDatum: WGS84; **Identification:** identifiedBy: Miloš Popović; **Event:** samplingProtocol: Observation; eventDate: 2013/08; year: 2013; month: 8; **Record Level:** type: Dataset; modified: WedWed/FebFeb/20162016; language: English; rightsHolder: Miloš Popović
- **Type status:** Other material. **Occurrence:** recordedBy: Miloš Popović; lifeStage: Adult; **Taxon:** kingdom: Animalia; phylum: Arthropoda; class: Insecta; order: Lepidoptera; family: Lycaenidae; genus: Phengaris; specificEpithet: teleius; scientificNameAuthorship: (Bergsträsser, 1779); **Location:** continent: Europe; country: Serbia; stateProvince: Vojvodina; decimalLatitude: 46.162965; decimalLongitude: 19.730587; geodeticDatum: WGS84; **Identification:** identifiedBy: Miloš Popović; **Event:** samplingProtocol: Observation; eventDate: 2013/08; year: 2013; month: 8; **Record Level:** type: Dataset; modified: WedWed/FebFeb/20162016; language: English; rightsHolder: Miloš Popović
- **Type status:** Other material. **Occurrence:** recordedBy: Miloš Popović; lifeStage: Adult; **Taxon:** kingdom: Animalia; phylum: Arthropoda; class: Insecta; order: Lepidoptera; family: Lycaenidae; genus: Phengaris; specificEpithet: teleius; scientificNameAuthorship: (Bergsträsser, 1779); **Location:** continent: Europe; country: Serbia; stateProvince: Vojvodina; decimalLatitude: 46.124781; decimalLongitude: 19.899969; geodeticDatum: WGS84; **Identification:** identifiedBy: Miloš Popović; **Event:** samplingProtocol: Observation; eventDate: 2013/08; year: 2013; month: 8; **Record Level:** type: Dataset; modified: WedWed/FebFeb/20162016; language: English; rightsHolder: Miloš Popović
- **Type status:** Other material. **Occurrence:** recordedBy: Miloš Popović; lifeStage: Adult; **Taxon:** kingdom: Animalia; phylum: Arthropoda; class: Insecta; order: Lepidoptera; family: Lycaenidae; genus: Phengaris; specificEpithet: teleius; scientificNameAuthorship: (Bergsträsser, 1779); **Location:** continent: Europe; country: Serbia; stateProvince: Vojvodina; decimalLatitude: 46.130651; decimalLongitude: 19.893778; geodeticDatum: WGS84; **Identification:** identifiedBy: Miloš Popović; **Event:** samplingProtocol: Observation; eventDate: 2013/08; year: 2013; month: 8; **Record Level:** type: Dataset; modified: WedWed/FebFeb/20162016; language: English; rightsHolder: Miloš Popović
- **Type status:** Other material. **Occurrence:** recordedBy: Miloš Popović; lifeStage: Adult; **Taxon:** kingdom: Animalia; phylum: Arthropoda; class: Insecta; order: Lepidoptera; family: Lycaenidae; genus: Phengaris; specificEpithet: teleius; scientificNameAuthorship: (Bergsträsser, 1779); **Location:** continent: Europe; country: Serbia; stateProvince: Vojvodina; decimalLatitude: 46.112924; decimalLongitude: 19.83855; geodeticDatum: WGS84; **Identification:** identifiedBy: Miloš Popović; **Event:** samplingProtocol: Observation; eventDate: 2013/08; year: 2013; month: 8; **Record Level:** type: Dataset; modified: WedWed/FebFeb/20162016; language: English; rightsHolder: Miloš Popović
- **Type status:** Other material. **Occurrence:** recordedBy: Miloš Popović; lifeStage: Adult; **Taxon:** kingdom: Animalia; phylum: Arthropoda; class: Insecta; order: Lepidoptera; family: Lycaenidae; genus: Phengaris; specificEpithet: teleius; scientificNameAuthorship: (Bergsträsser, 1779); **Location:** continent: Europe; country: Serbia; stateProvince: Vojvodina; decimalLatitude: 46.106345; decimalLongitude: 19.80586; geodeticDatum: WGS84; **Identification:** identifiedBy: Miloš Popović; **Event:** samplingProtocol: Observation; eventDate: 2013/08; year: 2013; month: 8; **Record Level:** type: Dataset; modified: WedWed/FebFeb/20162016; language: English; rightsHolder: Miloš Popović
- **Type status:** Other material. **Occurrence:** recordedBy: Miloš Popović; lifeStage: Adult; **Taxon:** kingdom: Animalia; phylum: Arthropoda; class: Insecta; order: Lepidoptera; family: Lycaenidae; genus: Phengaris; specificEpithet: teleius; scientificNameAuthorship: (Bergsträsser, 1779); **Location:** continent: Europe; country: Serbia; stateProvince: Vojvodina; decimalLatitude: 46.103968; decimalLongitude: 19.802332; geodeticDatum: WGS84; **Identification:** identifiedBy: Miloš Popović; **Event:** samplingProtocol: Observation; eventDate: 2013/08; year: 2013; month: 8; **Record Level:** type: Dataset; modified: WedWed/FebFeb/20162016; language: English; rightsHolder: Miloš Popović
- **Type status:** Other material. **Occurrence:** recordedBy: Miloš Popović; lifeStage: Adult; **Taxon:** kingdom: Animalia; phylum: Arthropoda; class: Insecta; order: Lepidoptera; family: Lycaenidae; genus: Phengaris; specificEpithet: teleius; scientificNameAuthorship: (Bergsträsser, 1779); **Location:** continent: Europe; country: Serbia; stateProvince: Vojvodina; decimalLatitude: 46.103595; decimalLongitude: 19.801877; geodeticDatum: WGS84; **Identification:** identifiedBy: Miloš Popović; **Event:** samplingProtocol: Observation; eventDate: 2013/08; year: 2013; month: 8; **Record Level:** type: Dataset; modified: WedWed/FebFeb/20162016; language: English; rightsHolder: Miloš Popović
- **Type status:** Other material. **Occurrence:** recordedBy: Miloš Popović; lifeStage: Adult; **Taxon:** kingdom: Animalia; phylum: Arthropoda; class: Insecta; order: Lepidoptera; family: Lycaenidae; genus: Phengaris; specificEpithet: teleius; scientificNameAuthorship: (Bergsträsser, 1779); **Location:** continent: Europe; country: Serbia; stateProvince: Vojvodina; decimalLatitude: 46.103757; decimalLongitude: 19.801363; geodeticDatum: WGS84; **Identification:** identifiedBy: Miloš Popović; **Event:** samplingProtocol: Observation; eventDate: 2013/08; year: 2013; month: 8; **Record Level:** type: Dataset; modified: WedWed/FebFeb/20162016; language: English; rightsHolder: Miloš Popović
- **Type status:** Other material. **Occurrence:** recordedBy: Miloš Popović; lifeStage: Adult; **Taxon:** kingdom: Animalia; phylum: Arthropoda; class: Insecta; order: Lepidoptera; family: Lycaenidae; genus: Phengaris; specificEpithet: teleius; scientificNameAuthorship: (Bergsträsser, 1779); **Location:** continent: Europe; country: Serbia; stateProvince: Vojvodina; decimalLatitude: 46.103826; decimalLongitude: 19.799965; geodeticDatum: WGS84; **Identification:** identifiedBy: Miloš Popović; **Event:** samplingProtocol: Observation; eventDate: 2013/08; year: 2013; month: 8; **Record Level:** type: Dataset; modified: WedWed/FebFeb/20162016; language: English; rightsHolder: Miloš Popović
- **Type status:** Other material. **Occurrence:** recordedBy: Miloš Popović; lifeStage: Adult; **Taxon:** kingdom: Animalia; phylum: Arthropoda; class: Insecta; order: Lepidoptera; family: Lycaenidae; genus: Phengaris; specificEpithet: teleius; scientificNameAuthorship: (Bergsträsser, 1779); **Location:** continent: Europe; country: Serbia; stateProvince: Vojvodina; decimalLatitude: 46.111654; decimalLongitude: 19.838993; geodeticDatum: WGS84; **Identification:** identifiedBy: Miloš Popović; **Event:** samplingProtocol: Observation; eventDate: 2013/08; year: 2013; month: 8; **Record Level:** type: Dataset; modified: WedWed/FebFeb/20162016; language: English; rightsHolder: Miloš Popović
- **Type status:** Other material. **Occurrence:** recordedBy: Miloš Popović; lifeStage: Adult; **Taxon:** kingdom: Animalia; phylum: Arthropoda; class: Insecta; order: Lepidoptera; family: Lycaenidae; genus: Phengaris; specificEpithet: teleius; scientificNameAuthorship: (Bergsträsser, 1779); **Location:** continent: Europe; country: Serbia; stateProvince: Vojvodina; decimalLatitude: 46.132745; decimalLongitude: 19.864977; geodeticDatum: WGS84; **Identification:** identifiedBy: Miloš Popović; **Event:** samplingProtocol: Observation; eventDate: 2013/08; year: 2013; month: 8; **Record Level:** type: Dataset; modified: WedWed/FebFeb/20162016; language: English; rightsHolder: Miloš Popović
- **Type status:** Other material. **Occurrence:** recordedBy: Miloš Popović; lifeStage: Adult; **Taxon:** kingdom: Animalia; phylum: Arthropoda; class: Insecta; order: Lepidoptera; family: Lycaenidae; genus: Phengaris; specificEpithet: teleius; scientificNameAuthorship: (Bergsträsser, 1779); **Location:** continent: Europe; country: Serbia; stateProvince: Vojvodina; decimalLatitude: 46.149406; decimalLongitude: 19.866331; geodeticDatum: WGS84; **Identification:** identifiedBy: Miloš Popović; **Event:** samplingProtocol: Observation; eventDate: 2013/08; year: 2013; month: 8; **Record Level:** type: Dataset; modified: WedWed/FebFeb/20162016; language: English; rightsHolder: Miloš Popović
- **Type status:** Other material. **Occurrence:** recordedBy: Miloš Popović; lifeStage: Adult; **Taxon:** kingdom: Animalia; phylum: Arthropoda; class: Insecta; order: Lepidoptera; family: Lycaenidae; genus: Phengaris; specificEpithet: teleius; scientificNameAuthorship: (Bergsträsser, 1779); **Location:** continent: Europe; country: Serbia; stateProvince: Vojvodina; decimalLatitude: 46.136546; decimalLongitude: 19.895377; geodeticDatum: WGS84; **Identification:** identifiedBy: Miloš Popović; **Event:** samplingProtocol: Observation; eventDate: 2013/08; year: 2013; month: 8; **Record Level:** type: Dataset; modified: WedWed/FebFeb/20162016; language: English; rightsHolder: Miloš Popović
- **Type status:** Other material. **Occurrence:** recordedBy: Miloš Popović; lifeStage: Adult; **Taxon:** kingdom: Animalia; phylum: Arthropoda; class: Insecta; order: Lepidoptera; family: Lycaenidae; genus: Phengaris; specificEpithet: teleius; scientificNameAuthorship: (Bergsträsser, 1779); **Location:** continent: Europe; country: Serbia; stateProvince: Vojvodina; decimalLatitude: 46.141199; decimalLongitude: 19.913294; geodeticDatum: WGS84; **Identification:** identifiedBy: Miloš Popović; **Event:** samplingProtocol: Observation; eventDate: 2013/08; year: 2013; month: 8; **Record Level:** type: Dataset; modified: WedWed/FebFeb/20162016; language: English; rightsHolder: Miloš Popović
- **Type status:** Other material. **Occurrence:** recordedBy: Miloš Popović; lifeStage: Adult; **Taxon:** kingdom: Animalia; phylum: Arthropoda; class: Insecta; order: Lepidoptera; family: Lycaenidae; genus: Phengaris; specificEpithet: teleius; scientificNameAuthorship: (Bergsträsser, 1779); **Location:** continent: Europe; country: Serbia; stateProvince: Vojvodina; decimalLatitude: 46.138319; decimalLongitude: 19.916135; geodeticDatum: WGS84; **Identification:** identifiedBy: Miloš Popović; **Event:** samplingProtocol: Observation; eventDate: 2013/08; year: 2013; month: 8; **Record Level:** type: Dataset; modified: WedWed/FebFeb/20162016; language: English; rightsHolder: Miloš Popović
- **Type status:** Other material. **Occurrence:** recordedBy: Miloš Popović; lifeStage: Adult; **Taxon:** kingdom: Animalia; phylum: Arthropoda; class: Insecta; order: Lepidoptera; family: Lycaenidae; genus: Phengaris; specificEpithet: teleius; scientificNameAuthorship: (Bergsträsser, 1779); **Location:** continent: Europe; country: Serbia; stateProvince: Vojvodina; decimalLatitude: 46.142506; decimalLongitude: 19.935001; geodeticDatum: WGS84; **Identification:** identifiedBy: Miloš Popović; **Event:** samplingProtocol: Observation; eventDate: 2013/08; year: 2013; month: 8; **Record Level:** type: Dataset; modified: WedWed/FebFeb/20162016; language: English; rightsHolder: Miloš Popović
- **Type status:** Other material. **Occurrence:** recordedBy: Miloš Popović; lifeStage: Adult; **Taxon:** kingdom: Animalia; phylum: Arthropoda; class: Insecta; order: Lepidoptera; family: Lycaenidae; genus: Phengaris; specificEpithet: teleius; scientificNameAuthorship: (Bergsträsser, 1779); **Location:** continent: Europe; country: Serbia; stateProvince: Vojvodina; decimalLatitude: 46.141279; decimalLongitude: 19.890007; geodeticDatum: WGS84; **Identification:** identifiedBy: Miloš Popović; **Event:** samplingProtocol: Observation; eventDate: 2013/08; year: 2013; month: 8; **Record Level:** type: Dataset; modified: WedWed/FebFeb/20162016; language: English; rightsHolder: Miloš Popović
- **Type status:** Other material. **Occurrence:** recordedBy: Miloš Popović; lifeStage: Adult; **Taxon:** kingdom: Animalia; phylum: Arthropoda; class: Insecta; order: Lepidoptera; family: Lycaenidae; genus: Phengaris; specificEpithet: teleius; scientificNameAuthorship: (Bergsträsser, 1779); **Location:** continent: Europe; country: Serbia; stateProvince: Vojvodina; decimalLatitude: 46.139543; decimalLongitude: 19.91937; geodeticDatum: WGS84; **Identification:** identifiedBy: Miloš Popović; **Event:** samplingProtocol: Observation; eventDate: 2013/08; year: 2013; month: 8; **Record Level:** type: Dataset; modified: WedWed/FebFeb/20162016; language: English; rightsHolder: Miloš Popović
- **Type status:** Other material. **Occurrence:** recordedBy: Miloš Popović; lifeStage: Adult; **Taxon:** kingdom: Animalia; phylum: Arthropoda; class: Insecta; order: Lepidoptera; family: Lycaenidae; genus: Phengaris; specificEpithet: teleius; scientificNameAuthorship: (Bergsträsser, 1779); **Location:** continent: Europe; country: Serbia; stateProvince: Vojvodina; decimalLatitude: 46.139254; decimalLongitude: 19.918326; geodeticDatum: WGS84; **Identification:** identifiedBy: Miloš Popović; **Event:** samplingProtocol: Observation; eventDate: 2013/08; year: 2013; month: 8; **Record Level:** type: Dataset; modified: WedWed/FebFeb/20162016; language: English; rightsHolder: Miloš Popović
- **Type status:** Other material. **Occurrence:** recordedBy: Miloš Popović; lifeStage: Adult; **Taxon:** kingdom: Animalia; phylum: Arthropoda; class: Insecta; order: Lepidoptera; family: Lycaenidae; genus: Phengaris; specificEpithet: teleius; scientificNameAuthorship: (Bergsträsser, 1779); **Location:** continent: Europe; country: Serbia; stateProvince: Vojvodina; decimalLatitude: 46.138871; decimalLongitude: 19.906927; geodeticDatum: WGS84; **Identification:** identifiedBy: Miloš Popović; **Event:** samplingProtocol: Observation; eventDate: 2013/08; year: 2013; month: 8; **Record Level:** type: Dataset; modified: WedWed/FebFeb/20162016; language: English; rightsHolder: Miloš Popović
- **Type status:** Other material. **Occurrence:** recordedBy: Miloš Popović; lifeStage: Adult; **Taxon:** kingdom: Animalia; phylum: Arthropoda; class: Insecta; order: Lepidoptera; family: Lycaenidae; genus: Phengaris; specificEpithet: teleius; scientificNameAuthorship: (Bergsträsser, 1779); **Location:** continent: Europe; country: Serbia; stateProvince: Vojvodina; decimalLatitude: 46.138867; decimalLongitude: 19.904966; geodeticDatum: WGS84; **Identification:** identifiedBy: Miloš Popović; **Event:** samplingProtocol: Observation; eventDate: 2013/08; year: 2013; month: 8; **Record Level:** type: Dataset; modified: WedWed/FebFeb/20162016; language: English; rightsHolder: Miloš Popović
- **Type status:** Other material. **Occurrence:** recordedBy: Miloš Popović; lifeStage: Adult; **Taxon:** kingdom: Animalia; phylum: Arthropoda; class: Insecta; order: Lepidoptera; family: Lycaenidae; genus: Phengaris; specificEpithet: teleius; scientificNameAuthorship: (Bergsträsser, 1779); **Location:** continent: Europe; country: Serbia; stateProvince: Vojvodina; decimalLatitude: 46.138654; decimalLongitude: 19.904645; geodeticDatum: WGS84; **Identification:** identifiedBy: Miloš Popović; **Event:** samplingProtocol: Observation; eventDate: 2013/08; year: 2013; month: 8; **Record Level:** type: Dataset; modified: WedWed/FebFeb/20162016; language: English; rightsHolder: Miloš Popović
- **Type status:** Other material. **Occurrence:** recordedBy: Miloš Popović; lifeStage: Adult; **Taxon:** kingdom: Animalia; phylum: Arthropoda; class: Insecta; order: Lepidoptera; family: Lycaenidae; genus: Phengaris; specificEpithet: teleius; scientificNameAuthorship: (Bergsträsser, 1779); **Location:** continent: Europe; country: Serbia; stateProvince: Vojvodina; decimalLatitude: 46.138175; decimalLongitude: 19.904383; geodeticDatum: WGS84; **Identification:** identifiedBy: Miloš Popović; **Event:** samplingProtocol: Observation; eventDate: 2013/08; year: 2013; month: 8; **Record Level:** type: Dataset; modified: WedWed/FebFeb/20162016; language: English; rightsHolder: Miloš Popović
- **Type status:** Other material. **Occurrence:** recordedBy: Miloš Popović; lifeStage: Adult; **Taxon:** kingdom: Animalia; phylum: Arthropoda; class: Insecta; order: Lepidoptera; family: Lycaenidae; genus: Phengaris; specificEpithet: teleius; scientificNameAuthorship: (Bergsträsser, 1779); **Location:** continent: Europe; country: Serbia; stateProvince: Vojvodina; decimalLatitude: 46.135235; decimalLongitude: 19.894115; geodeticDatum: WGS84; **Identification:** identifiedBy: Miloš Popović; **Event:** samplingProtocol: Observation; eventDate: 2013/08; year: 2013; month: 8; **Record Level:** type: Dataset; modified: WedWed/FebFeb/20162016; language: English; rightsHolder: Miloš Popović
- **Type status:** Other material. **Occurrence:** recordedBy: Miloš Popović; lifeStage: Adult; **Taxon:** kingdom: Animalia; phylum: Arthropoda; class: Insecta; order: Lepidoptera; family: Lycaenidae; genus: Phengaris; specificEpithet: teleius; scientificNameAuthorship: (Bergsträsser, 1779); **Location:** continent: Europe; country: Serbia; stateProvince: Vojvodina; decimalLatitude: 46.110357; decimalLongitude: 19.808614; geodeticDatum: WGS84; **Identification:** identifiedBy: Miloš Popović; **Event:** samplingProtocol: Observation; eventDate: 2013/08; year: 2013; month: 8; **Record Level:** type: Dataset; modified: WedWed/FebFeb/20162016; language: English; rightsHolder: Miloš Popović
- **Type status:** Other material. **Occurrence:** recordedBy: Miloš Popović; lifeStage: Adult; **Taxon:** kingdom: Animalia; phylum: Arthropoda; class: Insecta; order: Lepidoptera; family: Lycaenidae; genus: Phengaris; specificEpithet: teleius; scientificNameAuthorship: (Bergsträsser, 1779); **Location:** continent: Europe; country: Serbia; stateProvince: Vojvodina; decimalLatitude: 46.166978; decimalLongitude: 19.721403; geodeticDatum: WGS84; **Identification:** identifiedBy: Miloš Popović; **Event:** samplingProtocol: Observation; eventDate: 2013/08; year: 2013; month: 8; **Record Level:** type: Dataset; modified: WedWed/FebFeb/20162016; language: English; rightsHolder: Miloš Popović
- **Type status:** Other material. **Occurrence:** recordedBy: Miloš Popović; lifeStage: Adult; **Taxon:** kingdom: Animalia; phylum: Arthropoda; class: Insecta; order: Lepidoptera; family: Lycaenidae; genus: Phengaris; specificEpithet: teleius; scientificNameAuthorship: (Bergsträsser, 1779); **Location:** continent: Europe; country: Serbia; stateProvince: Vojvodina; decimalLatitude: 46.138126; decimalLongitude: 19.920623; geodeticDatum: WGS84; **Identification:** identifiedBy: Miloš Popović; **Event:** samplingProtocol: Observation; eventDate: 2013/08; year: 2013; month: 8; **Record Level:** type: Dataset; modified: WedWed/FebFeb/20162016; language: English; rightsHolder: Miloš Popović
- **Type status:** Other material. **Occurrence:** recordedBy: Miloš Popović; lifeStage: Adult; **Taxon:** kingdom: Animalia; phylum: Arthropoda; class: Insecta; order: Lepidoptera; family: Lycaenidae; genus: Phengaris; specificEpithet: teleius; scientificNameAuthorship: (Bergsträsser, 1779); **Location:** continent: Europe; country: Serbia; stateProvince: Vojvodina; decimalLatitude: 46.166433; decimalLongitude: 19.736874; geodeticDatum: WGS84; **Identification:** identifiedBy: Miloš Popović; **Event:** samplingProtocol: Observation; eventDate: 2013/08; year: 2013; month: 8; **Record Level:** type: Dataset; modified: WedWed/FebFeb/20162016; language: English; rightsHolder: Miloš Popović
- **Type status:** Other material. **Occurrence:** recordedBy: Miloš Popović; lifeStage: Adult; **Taxon:** kingdom: Animalia; phylum: Arthropoda; class: Insecta; order: Lepidoptera; family: Lycaenidae; genus: Phengaris; specificEpithet: teleius; scientificNameAuthorship: (Bergsträsser, 1779); **Location:** continent: Europe; country: Serbia; stateProvince: Vojvodina; decimalLatitude: 46.16917; decimalLongitude: 19.733129; geodeticDatum: WGS84; **Identification:** identifiedBy: Miloš Popović; **Event:** samplingProtocol: Observation; eventDate: 2013/08; year: 2013; month: 8; **Record Level:** type: Dataset; modified: WedWed/FebFeb/20162016; language: English; rightsHolder: Miloš Popović
- **Type status:** Other material. **Occurrence:** recordedBy: Miloš Popović; lifeStage: Adult; **Taxon:** kingdom: Animalia; phylum: Arthropoda; class: Insecta; order: Lepidoptera; family: Lycaenidae; genus: Phengaris; specificEpithet: teleius; scientificNameAuthorship: (Bergsträsser, 1779); **Location:** continent: Europe; country: Serbia; stateProvince: Vojvodina; decimalLatitude: 46.167108; decimalLongitude: 19.730292; geodeticDatum: WGS84; **Identification:** identifiedBy: Miloš Popović; **Event:** samplingProtocol: Observation; eventDate: 2013/08; year: 2013; month: 8; **Record Level:** type: Dataset; modified: WedWed/FebFeb/20162016; language: English; rightsHolder: Miloš Popović
- **Type status:** Other material. **Occurrence:** recordedBy: Miloš Popović; lifeStage: Adult; **Taxon:** kingdom: Animalia; phylum: Arthropoda; class: Insecta; order: Lepidoptera; family: Lycaenidae; genus: Phengaris; specificEpithet: teleius; scientificNameAuthorship: (Bergsträsser, 1779); **Location:** continent: Europe; country: Serbia; stateProvince: Vojvodina; decimalLatitude: 46.181499; decimalLongitude: 19.691777; geodeticDatum: WGS84; **Identification:** identifiedBy: Miloš Popović; **Event:** samplingProtocol: Observation; eventDate: 2013/08; year: 2013; month: 8; **Record Level:** type: Dataset; modified: WedWed/FebFeb/20162016; language: English; rightsHolder: Miloš Popović
- **Type status:** Other material. **Occurrence:** recordedBy: Miloš Popović; lifeStage: Adult; **Taxon:** kingdom: Animalia; phylum: Arthropoda; class: Insecta; order: Lepidoptera; family: Lycaenidae; genus: Phengaris; specificEpithet: teleius; scientificNameAuthorship: (Bergsträsser, 1779); **Location:** continent: Europe; country: Serbia; stateProvince: Vojvodina; decimalLatitude: 46.143362; decimalLongitude: 19.911539; geodeticDatum: WGS84; **Identification:** identifiedBy: Miloš Popović; **Event:** samplingProtocol: Observation; eventDate: 2013/08; year: 2013; month: 8; **Record Level:** type: Dataset; modified: WedWed/FebFeb/20162016; language: English; rightsHolder: Miloš Popović
- **Type status:** Other material. **Occurrence:** recordedBy: Miloš Popović; lifeStage: Adult; **Taxon:** kingdom: Animalia; phylum: Arthropoda; class: Insecta; order: Lepidoptera; family: Lycaenidae; genus: Phengaris; specificEpithet: teleius; scientificNameAuthorship: (Bergsträsser, 1779); **Location:** continent: Europe; country: Serbia; stateProvince: Vojvodina; decimalLatitude: 46.14779; decimalLongitude: 19.908017; geodeticDatum: WGS84; **Identification:** identifiedBy: Miloš Popović; **Event:** samplingProtocol: Observation; eventDate: 2013/08; year: 2013; month: 8; **Record Level:** type: Dataset; modified: WedWed/FebFeb/20162016; language: English; rightsHolder: Miloš Popović
- **Type status:** Other material. **Occurrence:** recordedBy: Miloš Popović; lifeStage: Adult; **Taxon:** kingdom: Animalia; phylum: Arthropoda; class: Insecta; order: Lepidoptera; family: Lycaenidae; genus: Phengaris; specificEpithet: teleius; scientificNameAuthorship: (Bergsträsser, 1779); **Location:** continent: Europe; country: Serbia; stateProvince: Vojvodina; decimalLatitude: 46.103589; decimalLongitude: 19.800948; geodeticDatum: WGS84; **Identification:** identifiedBy: Miloš Popović; **Event:** samplingProtocol: Observation; eventDate: 2013/08; year: 2013; month: 8; **Record Level:** type: Dataset; modified: WedWed/FebFeb/20162016; language: English; rightsHolder: Miloš Popović
- **Type status:** Other material. **Occurrence:** recordedBy: Miloš Popović; lifeStage: Adult; **Taxon:** kingdom: Animalia; phylum: Arthropoda; class: Insecta; order: Lepidoptera; family: Lycaenidae; genus: Phengaris; specificEpithet: teleius; scientificNameAuthorship: (Bergsträsser, 1779); **Location:** continent: Europe; country: Serbia; stateProvince: Vojvodina; decimalLatitude: 46.103077; decimalLongitude: 19.800767; geodeticDatum: WGS84; **Identification:** identifiedBy: Miloš Popović; **Event:** samplingProtocol: Observation; eventDate: 2013/08; year: 2013; month: 8; **Record Level:** type: Dataset; modified: WedWed/FebFeb/20162016; language: English; rightsHolder: Miloš Popović
- **Type status:** Other material. **Occurrence:** recordedBy: Miloš Popović; lifeStage: Adult; **Taxon:** kingdom: Animalia; phylum: Arthropoda; class: Insecta; order: Lepidoptera; family: Lycaenidae; genus: Phengaris; specificEpithet: teleius; scientificNameAuthorship: (Bergsträsser, 1779); **Location:** continent: Europe; country: Serbia; stateProvince: Vojvodina; decimalLatitude: 46.111453; decimalLongitude: 19.789816; geodeticDatum: WGS84; **Identification:** identifiedBy: Miloš Popović; **Event:** samplingProtocol: Observation; eventDate: 2014/07/19; year: 2014; month: 7; day: 19; **Record Level:** type: Dataset; modified: WedWed/FebFeb/20162016; language: English; rightsHolder: Miloš Popović
- **Type status:** Other material. **Occurrence:** recordedBy: Miloš Popović; lifeStage: Adult; **Taxon:** kingdom: Animalia; phylum: Arthropoda; class: Insecta; order: Lepidoptera; family: Lycaenidae; genus: Phengaris; specificEpithet: teleius; scientificNameAuthorship: (Bergsträsser, 1779); **Location:** continent: Europe; country: Serbia; stateProvince: Vojvodina; decimalLatitude: 46.111743; decimalLongitude: 19.789677; geodeticDatum: WGS84; **Identification:** identifiedBy: Miloš Popović; **Event:** samplingProtocol: Observation; eventDate: 2015/08/03; year: 2015; month: 8; day: 3; **Record Level:** type: Dataset; modified: WedWed/FebFeb/20162016; language: English; rightsHolder: Miloš Popović
- **Type status:** Other material. **Occurrence:** recordedBy: Miloš Popović; lifeStage: Adult; **Taxon:** kingdom: Animalia; phylum: Arthropoda; class: Insecta; order: Lepidoptera; family: Lycaenidae; genus: Lycaena; specificEpithet: dispar; scientificNameAuthorship: (Haworth, 1802); **Location:** continent: Europe; country: Serbia; stateProvince: Vojvodina; decimalLatitude: 46.111743; decimalLongitude: 19.789677; geodeticDatum: WGS84; **Identification:** identifiedBy: Miloš Popović; **Event:** samplingProtocol: Observation; eventDate: 2015/08/03; year: 2015; month: 8; day: 3; **Record Level:** type: Dataset; modified: WedWed/FebFeb/20162016; language: English; rightsHolder: Miloš Popović
- **Type status:** Other material. **Occurrence:** recordedBy: Miloš Popović; lifeStage: Adult; **Taxon:** kingdom: Animalia; phylum: Arthropoda; class: Insecta; order: Lepidoptera; family: Nymphalidae; genus: Coenonympha; specificEpithet: glycerion; scientificNameAuthorship: (Borkhausen, 1788); **Location:** continent: Europe; country: Serbia; stateProvince: Vojvodina; decimalLatitude: 46.111743; decimalLongitude: 19.789677; geodeticDatum: WGS84; **Identification:** identifiedBy: Miloš Popović; **Event:** samplingProtocol: Observation; eventDate: 2015/08/03; year: 2015; month: 8; day: 3; **Record Level:** type: Dataset; modified: WedWed/FebFeb/20162016; language: English; rightsHolder: Miloš Popović
- **Type status:** Other material. **Occurrence:** recordedBy: Miloš Popović, Martina Šašić; lifeStage: Adult; **Taxon:** kingdom: Animalia; phylum: Arthropoda; class: Insecta; order: Lepidoptera; family: Lycaenidae; genus: Polyommatus; specificEpithet: icarus; scientificNameAuthorship: (Rottemburg, 1775); **Location:** continent: Europe; country: Hungary; decimalLatitude: 46.148631; decimalLongitude: 19.777267; geodeticDatum: WGS84; **Identification:** identifiedBy: Miloš Popović, Martina Šašić; **Event:** samplingProtocol: Observation; eventDate: 2015/08/06; year: 2015; month: 8; day: 6; **Record Level:** type: Dataset; modified: WedWed/FebFeb/20162016; language: English; rightsHolder: Miloš Popović
- **Type status:** Other material. **Occurrence:** recordedBy: Miloš Popović, Martina Šašić; lifeStage: Adult; **Taxon:** kingdom: Animalia; phylum: Arthropoda; class: Insecta; order: Lepidoptera; family: Nymphalidae; genus: Argynnis; specificEpithet: pandora; scientificNameAuthorship: (Denis & Schiffermüller, 1775); **Location:** continent: Europe; country: Hungary; decimalLatitude: 46.148631; decimalLongitude: 19.777267; geodeticDatum: WGS84; **Identification:** identifiedBy: Miloš Popović, Martina Šašić; **Event:** samplingProtocol: Observation; eventDate: 2015/08/06; year: 2015; month: 8; day: 6; **Record Level:** type: Dataset; modified: WedWed/FebFeb/20162016; language: English; rightsHolder: Miloš Popović
- **Type status:** Other material. **Occurrence:** recordedBy: Miloš Popović, Martina Šašić; lifeStage: Adult; **Taxon:** kingdom: Animalia; phylum: Arthropoda; class: Insecta; order: Lepidoptera; family: Hesperiidae; genus: Hesperia; specificEpithet: comma; scientificNameAuthorship: (Linnaeus, 1758); **Location:** continent: Europe; country: Hungary; decimalLatitude: 46.148631; decimalLongitude: 19.777267; geodeticDatum: WGS84; **Identification:** identifiedBy: Miloš Popović, Martina Šašić; **Event:** samplingProtocol: Observation; eventDate: 2015/08/06; year: 2015; month: 8; day: 6; **Record Level:** type: Dataset; modified: WedWed/FebFeb/20162016; language: English; rightsHolder: Miloš Popović
- **Type status:** Other material. **Occurrence:** recordedBy: Miloš Popović, Martina Šašić; lifeStage: Adult; **Taxon:** kingdom: Animalia; phylum: Arthropoda; class: Insecta; order: Lepidoptera; family: Papilionidae; genus: Papilio; specificEpithet: machaon; scientificNameAuthorship: Linnaeus, 1758; **Location:** continent: Europe; country: Hungary; decimalLatitude: 46.148631; decimalLongitude: 19.777267; geodeticDatum: WGS84; **Identification:** identifiedBy: Miloš Popović, Martina Šašić; **Event:** samplingProtocol: Observation; eventDate: 2015/08/06; year: 2015; month: 8; day: 6; **Record Level:** type: Dataset; modified: WedWed/FebFeb/20162016; language: English; rightsHolder: Miloš Popović
- **Type status:** Other material. **Occurrence:** recordedBy: Miloš Popović, Martina Šašić; lifeStage: Adult; **Taxon:** kingdom: Animalia; phylum: Arthropoda; class: Insecta; order: Lepidoptera; family: Lycaenidae; genus: Polyommatus; specificEpithet: coridon; scientificNameAuthorship: (Poda, 1761); **Location:** continent: Europe; country: Hungary; decimalLatitude: 46.148631; decimalLongitude: 19.777267; geodeticDatum: WGS84; **Identification:** identifiedBy: Miloš Popović, Martina Šašić; **Event:** samplingProtocol: Observation; eventDate: 2015/08/06; year: 2015; month: 8; day: 6; **Record Level:** type: Dataset; modified: WedWed/FebFeb/20162016; language: English; rightsHolder: Miloš Popović
- **Type status:** Other material. **Occurrence:** recordedBy: Miloš Popović, Martina Šašić; lifeStage: Adult; **Taxon:** kingdom: Animalia; phylum: Arthropoda; class: Insecta; order: Lepidoptera; family: Lycaenidae; genus: Plebejus; specificEpithet: argus; scientificNameAuthorship: (Linnaeus, 1758); **Location:** continent: Europe; country: Hungary; decimalLatitude: 46.148631; decimalLongitude: 19.777267; geodeticDatum: WGS84; **Identification:** identifiedBy: Miloš Popović, Martina Šašić; **Event:** samplingProtocol: Observation; eventDate: 2015/08/06; year: 2015; month: 8; day: 6; **Record Level:** type: Dataset; modified: WedWed/FebFeb/20162016; language: English; rightsHolder: Miloš Popović
- **Type status:** Other material. **Occurrence:** recordedBy: Miloš Popović, Martina Šašić; lifeStage: Adult; **Taxon:** kingdom: Animalia; phylum: Arthropoda; class: Insecta; order: Lepidoptera; family: Lycaenidae; genus: Lycaena; specificEpithet: phlaeas; scientificNameAuthorship: (Linnaeus, 1761); **Location:** continent: Europe; country: Hungary; decimalLatitude: 46.148631; decimalLongitude: 19.777267; geodeticDatum: WGS84; **Identification:** identifiedBy: Miloš Popović, Martina Šašić; **Event:** samplingProtocol: Observation; eventDate: 2015/08/06; year: 2015; month: 8; day: 6; **Record Level:** type: Dataset; modified: WedWed/FebFeb/20162016; language: English; rightsHolder: Miloš Popović
- **Type status:** Other material. **Occurrence:** recordedBy: Miloš Popović, Martina Šašić; lifeStage: Adult; **Taxon:** kingdom: Animalia; phylum: Arthropoda; class: Insecta; order: Lepidoptera; family: Nymphalidae; genus: Melitaea; specificEpithet: phoebe; scientificNameAuthorship: (Denis & Schiffermüller, 1775); **Location:** continent: Europe; country: Hungary; decimalLatitude: 46.148631; decimalLongitude: 19.777267; geodeticDatum: WGS84; **Identification:** identifiedBy: Miloš Popović, Martina Šašić; **Event:** samplingProtocol: Observation; eventDate: 2015/08/06; year: 2015; month: 8; day: 6; **Record Level:** type: Dataset; modified: WedWed/FebFeb/20162016; language: English; rightsHolder: Miloš Popović
- **Type status:** Other material. **Occurrence:** recordedBy: Miloš Popović, Martina Šašić; lifeStage: Adult; **Taxon:** kingdom: Animalia; phylum: Arthropoda; class: Insecta; order: Lepidoptera; family: Nymphalidae; genus: Maniola; specificEpithet: jurtina; scientificNameAuthorship: (Linnaeus, 1758); **Location:** continent: Europe; country: Hungary; decimalLatitude: 46.148631; decimalLongitude: 19.777267; geodeticDatum: WGS84; **Identification:** identifiedBy: Miloš Popović, Martina Šašić; **Event:** samplingProtocol: Observation; eventDate: 2015/08/06; year: 2015; month: 8; day: 6; **Record Level:** type: Dataset; modified: WedWed/FebFeb/20162016; language: English; rightsHolder: Miloš Popović
- **Type status:** Other material. **Occurrence:** recordedBy: Miloš Popović, Martina Šašić; lifeStage: Adult; **Taxon:** kingdom: Animalia; phylum: Arthropoda; class: Insecta; order: Lepidoptera; family: Nymphalidae; genus: Araschnia; specificEpithet: levana; scientificNameAuthorship: (Linnaeus, 1758); **Location:** continent: Europe; country: Hungary; decimalLatitude: 46.148631; decimalLongitude: 19.777267; geodeticDatum: WGS84; **Identification:** identifiedBy: Miloš Popović, Martina Šašić; **Event:** samplingProtocol: Observation; eventDate: 2015/08/06; year: 2015; month: 8; day: 6; **Record Level:** type: Dataset; modified: WedWed/FebFeb/20162016; language: English; rightsHolder: Miloš Popović
- **Type status:** Other material. **Occurrence:** recordedBy: Miloš Popović, Martina Šašić; lifeStage: Adult; **Taxon:** kingdom: Animalia; phylum: Arthropoda; class: Insecta; order: Lepidoptera; family: Lycaenidae; genus: Celastrina; specificEpithet: argiolus; scientificNameAuthorship: (Linnaeus, 1758); **Location:** continent: Europe; country: Hungary; decimalLatitude: 46.148631; decimalLongitude: 19.777267; geodeticDatum: WGS84; **Identification:** identifiedBy: Miloš Popović, Martina Šašić; **Event:** samplingProtocol: Observation; eventDate: 2015/08/06; year: 2015; month: 8; day: 6; **Record Level:** type: Dataset; modified: WedWed/FebFeb/20162016; language: English; rightsHolder: Miloš Popović
- **Type status:** Other material. **Occurrence:** recordedBy: Miloš Popović, Martina Šašić; lifeStage: Adult; **Taxon:** kingdom: Animalia; phylum: Arthropoda; class: Insecta; order: Lepidoptera; family: Nymphalidae; genus: Coenonympha; specificEpithet: glycerion; scientificNameAuthorship: (Borkhausen, 1788); **Location:** continent: Europe; country: Hungary; decimalLatitude: 46.148631; decimalLongitude: 19.777267; geodeticDatum: WGS84; **Identification:** identifiedBy: Miloš Popović, Martina Šašić; **Event:** samplingProtocol: Observation; eventDate: 2015/08/06; year: 2015; month: 8; day: 6; **Record Level:** type: Dataset; modified: WedWed/FebFeb/20162016; language: English; rightsHolder: Miloš Popović
- **Type status:** Other material. **Occurrence:** recordedBy: Miloš Popović, Martina Šašić; lifeStage: Adult; **Taxon:** kingdom: Animalia; phylum: Arthropoda; class: Insecta; order: Lepidoptera; family: Pieridae; genus: Colias; specificEpithet: erate; scientificNameAuthorship: (Esper, 1805); **Location:** continent: Europe; country: Hungary; decimalLatitude: 46.150242; decimalLongitude: 19.776972; geodeticDatum: WGS84; **Identification:** identifiedBy: Miloš Popović, Martina Šašić; **Event:** samplingProtocol: Observation; eventDate: 2015/08/06; year: 2015; month: 8; day: 6; **Record Level:** type: Dataset; modified: WedWed/FebFeb/20162016; language: English; rightsHolder: Miloš Popović
- **Type status:** Other material. **Occurrence:** recordedBy: Miloš Popović, Martina Šašić; lifeStage: Adult; **Taxon:** kingdom: Animalia; phylum: Arthropoda; class: Insecta; order: Lepidoptera; family: Nymphalidae; genus: Minois; specificEpithet: dryas; scientificNameAuthorship: (Scopoli, 1763); **Location:** continent: Europe; country: Hungary; decimalLatitude: 46.150242; decimalLongitude: 19.776972; geodeticDatum: WGS84; **Identification:** identifiedBy: Miloš Popović, Martina Šašić; **Event:** samplingProtocol: Observation; eventDate: 2015/08/06; year: 2015; month: 8; day: 6; **Record Level:** type: Dataset; modified: WedWed/FebFeb/20162016; language: English; rightsHolder: Miloš Popović
- **Type status:** Other material. **Occurrence:** recordedBy: Miloš Popović, Martina Šašić; lifeStage: Adult; **Taxon:** kingdom: Animalia; phylum: Arthropoda; class: Insecta; order: Lepidoptera; family: Lycaenidae; genus: Lycaena; specificEpithet: dispar; scientificNameAuthorship: (Haworth, 1802); **Location:** continent: Europe; country: Hungary; decimalLatitude: 46.150242; decimalLongitude: 19.776972; geodeticDatum: WGS84; **Identification:** identifiedBy: Miloš Popović, Martina Šašić; **Event:** samplingProtocol: Observation; eventDate: 2015/08/06; year: 2015; month: 8; day: 6; **Record Level:** type: Dataset; modified: WedWed/FebFeb/20162016; language: English; rightsHolder: Miloš Popović
- **Type status:** Other material. **Occurrence:** recordedBy: Miloš Popović, Martina Šašić; lifeStage: Adult; **Taxon:** kingdom: Animalia; phylum: Arthropoda; class: Insecta; order: Lepidoptera; family: Lycaenidae; genus: Phengaris; specificEpithet: teleius; scientificNameAuthorship: (Bergsträsser, 1779); **Location:** continent: Europe; country: Hungary; decimalLatitude: 46.150242; decimalLongitude: 19.776972; geodeticDatum: WGS84; **Identification:** identifiedBy: Miloš Popović, Martina Šašić; **Event:** samplingProtocol: Observation; eventDate: 2015/08/06; year: 2015; month: 8; day: 6; **Record Level:** type: Dataset; modified: WedWed/FebFeb/20162016; language: English; rightsHolder: Miloš Popović
- **Type status:** Other material. **Occurrence:** recordedBy: Miloš Popović, Martina Šašić; lifeStage: Adult; **Taxon:** kingdom: Animalia; phylum: Arthropoda; class: Insecta; order: Lepidoptera; family: Lycaenidae; genus: Lycaena; specificEpithet: phlaeas; scientificNameAuthorship: (Linnaeus, 1761); **Location:** continent: Europe; country: Hungary; decimalLatitude: 46.150242; decimalLongitude: 19.776972; geodeticDatum: WGS84; **Identification:** identifiedBy: Miloš Popović, Martina Šašić; **Event:** samplingProtocol: Observation; eventDate: 2015/08/06; year: 2015; month: 8; day: 6; **Record Level:** type: Dataset; modified: WedWed/FebFeb/20162016; language: English; rightsHolder: Miloš Popović
- **Type status:** Other material. **Occurrence:** recordedBy: Miloš Popović, Martina Šašić; lifeStage: Adult; **Taxon:** kingdom: Animalia; phylum: Arthropoda; class: Insecta; order: Lepidoptera; family: Hesperiidae; genus: Hesperia; specificEpithet: comma; scientificNameAuthorship: (Linnaeus, 1758); **Location:** continent: Europe; country: Hungary; decimalLatitude: 46.150242; decimalLongitude: 19.776972; geodeticDatum: WGS84; **Identification:** identifiedBy: Miloš Popović, Martina Šašić; **Event:** samplingProtocol: Observation; eventDate: 2015/08/06; year: 2015; month: 8; day: 6; **Record Level:** type: Dataset; modified: WedWed/FebFeb/20162016; language: English; rightsHolder: Miloš Popović
- **Type status:** Other material. **Occurrence:** recordedBy: Miloš Popović, Martina Šašić; lifeStage: Adult; **Taxon:** kingdom: Animalia; phylum: Arthropoda; class: Insecta; order: Lepidoptera; family: Lycaenidae; genus: Polyommatus; specificEpithet: icarus; scientificNameAuthorship: (Rottemburg, 1775); **Location:** continent: Europe; country: Hungary; decimalLatitude: 46.150242; decimalLongitude: 19.776972; geodeticDatum: WGS84; **Identification:** identifiedBy: Miloš Popović, Martina Šašić; **Event:** samplingProtocol: Observation; eventDate: 2015/08/06; year: 2015; month: 8; day: 6; **Record Level:** type: Dataset; modified: WedWed/FebFeb/20162016; language: English; rightsHolder: Miloš Popović
- **Type status:** Other material. **Occurrence:** recordedBy: Miloš Popović, Martina Šašić; lifeStage: Adult; **Taxon:** kingdom: Animalia; phylum: Arthropoda; class: Insecta; order: Lepidoptera; family: Lycaenidae; genus: Polyommatus; specificEpithet: coridon; scientificNameAuthorship: (Poda, 1761); **Location:** continent: Europe; country: Hungary; decimalLatitude: 46.150242; decimalLongitude: 19.776972; geodeticDatum: WGS84; **Identification:** identifiedBy: Miloš Popović, Martina Šašić; **Event:** samplingProtocol: Observation; eventDate: 2015/08/06; year: 2015; month: 8; day: 6; **Record Level:** type: Dataset; modified: WedWed/FebFeb/20162016; language: English; rightsHolder: Miloš Popović
- **Type status:** Other material. **Occurrence:** recordedBy: Miloš Popović, Martina Šašić; lifeStage: Adult; **Taxon:** kingdom: Animalia; phylum: Arthropoda; class: Insecta; order: Lepidoptera; family: Nymphalidae; genus: Maniola; specificEpithet: jurtina; scientificNameAuthorship: (Linnaeus, 1758); **Location:** continent: Europe; country: Hungary; decimalLatitude: 46.150242; decimalLongitude: 19.776972; geodeticDatum: WGS84; **Identification:** identifiedBy: Miloš Popović, Martina Šašić; **Event:** samplingProtocol: Observation; eventDate: 2015/08/06; year: 2015; month: 8; day: 6; **Record Level:** type: Dataset; modified: WedWed/FebFeb/20162016; language: English; rightsHolder: Miloš Popović
- **Type status:** Other material. **Occurrence:** recordedBy: Miloš Popović, Martina Šašić; lifeStage: Adult; **Taxon:** kingdom: Animalia; phylum: Arthropoda; class: Insecta; order: Lepidoptera; family: Nymphalidae; genus: Coenonympha; specificEpithet: glycerion; scientificNameAuthorship: (Borkhausen, 1788); **Location:** continent: Europe; country: Hungary; decimalLatitude: 46.19816; decimalLongitude: 19.830556; geodeticDatum: WGS84; **Identification:** identifiedBy: Miloš Popović, Martina Šašić; **Event:** samplingProtocol: Observation; eventDate: 2015/08/06; year: 2015; month: 8; day: 6; **Record Level:** type: Dataset; modified: WedWed/FebFeb/20162016; language: English; rightsHolder: Miloš Popović
- **Type status:** Other material. **Occurrence:** recordedBy: Miloš Popović, Martina Šašić; lifeStage: Adult; **Taxon:** kingdom: Animalia; phylum: Arthropoda; class: Insecta; order: Lepidoptera; family: Lycaenidae; genus: Lycaena; specificEpithet: phlaeas; scientificNameAuthorship: (Linnaeus, 1761); **Location:** continent: Europe; country: Hungary; decimalLatitude: 46.19816; decimalLongitude: 19.830556; geodeticDatum: WGS84; **Identification:** identifiedBy: Miloš Popović, Martina Šašić; **Event:** samplingProtocol: Observation; eventDate: 2015/08/06; year: 2015; month: 8; day: 6; **Record Level:** type: Dataset; modified: WedWed/FebFeb/20162016; language: English; rightsHolder: Miloš Popović
- **Type status:** Other material. **Occurrence:** recordedBy: Miloš Popović, Martina Šašić; lifeStage: Adult; **Taxon:** kingdom: Animalia; phylum: Arthropoda; class: Insecta; order: Lepidoptera; family: Pieridae; genus: Leptidea; specificEpithet: sinapis; scientificNameAuthorship: (Linnaeus, 1758); **Location:** continent: Europe; country: Hungary; decimalLatitude: 46.19816; decimalLongitude: 19.830556; geodeticDatum: WGS84; **Identification:** identifiedBy: Miloš Popović, Martina Šašić; **Event:** samplingProtocol: Observation; eventDate: 2015/08/06; year: 2015; month: 8; day: 6; **Record Level:** type: Dataset; modified: WedWed/FebFeb/20162016; language: English; rightsHolder: Miloš Popović
- **Type status:** Other material. **Occurrence:** recordedBy: Miloš Popović, Martina Šašić; lifeStage: Adult; **Taxon:** kingdom: Animalia; phylum: Arthropoda; class: Insecta; order: Lepidoptera; family: Lycaenidae; genus: Lycaena; specificEpithet: dispar; scientificNameAuthorship: (Haworth, 1802); **Location:** continent: Europe; country: Hungary; decimalLatitude: 46.19816; decimalLongitude: 19.830556; geodeticDatum: WGS84; **Identification:** identifiedBy: Miloš Popović, Martina Šašić; **Event:** samplingProtocol: Observation; eventDate: 2015/08/06; year: 2015; month: 8; day: 6; **Record Level:** type: Dataset; modified: WedWed/FebFeb/20162016; language: English; rightsHolder: Miloš Popović
- **Type status:** Other material. **Occurrence:** recordedBy: Miloš Popović, Martina Šašić; lifeStage: Adult; **Taxon:** kingdom: Animalia; phylum: Arthropoda; class: Insecta; order: Lepidoptera; family: Lycaenidae; genus: Phengaris; specificEpithet: teleius; scientificNameAuthorship: (Bergsträsser, 1779); **Location:** continent: Europe; country: Hungary; decimalLatitude: 46.19816; decimalLongitude: 19.830556; geodeticDatum: WGS84; **Identification:** identifiedBy: Miloš Popović, Martina Šašić; **Event:** samplingProtocol: Observation; eventDate: 2015/08/06; year: 2015; month: 8; day: 6; **Record Level:** type: Dataset; modified: WedWed/FebFeb/20162016; language: English; rightsHolder: Miloš Popović
- **Type status:** Other material. **Occurrence:** recordedBy: Miloš Popović, Martina Šašić; lifeStage: Adult; **Taxon:** kingdom: Animalia; phylum: Arthropoda; class: Insecta; order: Lepidoptera; family: Lycaenidae; genus: Plebejus; specificEpithet: argyrognomon; scientificNameAuthorship: (Bergsträsser, 1779); **Location:** continent: Europe; country: Hungary; decimalLatitude: 46.19816; decimalLongitude: 19.830556; geodeticDatum: WGS84; **Identification:** identifiedBy: Miloš Popović, Martina Šašić; **Event:** samplingProtocol: Observation; eventDate: 2015/08/06; year: 2015; month: 8; day: 6; **Record Level:** type: Dataset; modified: WedWed/FebFeb/20162016; language: English; rightsHolder: Miloš Popović
- **Type status:** Other material. **Occurrence:** recordedBy: Miloš Popović, Martina Šašić; lifeStage: Adult; **Taxon:** kingdom: Animalia; phylum: Arthropoda; class: Insecta; order: Lepidoptera; family: Lycaenidae; genus: Polyommatus; specificEpithet: icarus; scientificNameAuthorship: (Rottemburg, 1775); **Location:** continent: Europe; country: Hungary; decimalLatitude: 46.19816; decimalLongitude: 19.830556; geodeticDatum: WGS84; **Identification:** identifiedBy: Miloš Popović, Martina Šašić; **Event:** samplingProtocol: Observation; eventDate: 2015/08/06; year: 2015; month: 8; day: 6; **Record Level:** type: Dataset; modified: WedWed/FebFeb/20162016; language: English; rightsHolder: Miloš Popović
- **Type status:** Other material. **Occurrence:** recordedBy: Miloš Popović, Martina Šašić; lifeStage: Adult; **Taxon:** kingdom: Animalia; phylum: Arthropoda; class: Insecta; order: Lepidoptera; family: Pieridae; genus: Colias; specificEpithet: erate; scientificNameAuthorship: (Esper, 1805); **Location:** continent: Europe; country: Hungary; decimalLatitude: 46.19816; decimalLongitude: 19.830556; geodeticDatum: WGS84; **Identification:** identifiedBy: Miloš Popović, Martina Šašić; **Event:** samplingProtocol: Observation; eventDate: 2015/08/06; year: 2015; month: 8; day: 6; **Record Level:** type: Dataset; modified: WedWed/FebFeb/20162016; language: English; rightsHolder: Miloš Popović
- **Type status:** Other material. **Occurrence:** recordedBy: Miloš Popović, Martina Šašić; lifeStage: Adult; **Taxon:** kingdom: Animalia; phylum: Arthropoda; class: Insecta; order: Lepidoptera; family: Nymphalidae; genus: Issoria; specificEpithet: lathonia; scientificNameAuthorship: (Linnaeus, 1758); **Location:** continent: Europe; country: Hungary; decimalLatitude: 46.19816; decimalLongitude: 19.830556; geodeticDatum: WGS84; **Identification:** identifiedBy: Miloš Popović, Martina Šašić; **Event:** samplingProtocol: Observation; eventDate: 2015/08/06; year: 2015; month: 8; day: 6; **Record Level:** type: Dataset; modified: WedWed/FebFeb/20162016; language: English; rightsHolder: Miloš Popović
- **Type status:** Other material. **Occurrence:** recordedBy: Miloš Popović, Martina Šašić; lifeStage: Adult; **Taxon:** kingdom: Animalia; phylum: Arthropoda; class: Insecta; order: Lepidoptera; family: Lycaenidae; genus: Lycaena; specificEpithet: tityrus; scientificNameAuthorship: (Poda, 1761); **Location:** continent: Europe; country: Hungary; decimalLatitude: 46.19816; decimalLongitude: 19.830556; geodeticDatum: WGS84; **Identification:** identifiedBy: Miloš Popović, Martina Šašić; **Event:** samplingProtocol: Observation; eventDate: 2015/08/06; year: 2015; month: 8; day: 6; **Record Level:** type: Dataset; modified: WedWed/FebFeb/20162016; language: English; rightsHolder: Miloš Popović
- **Type status:** Other material. **Occurrence:** recordedBy: Miloš Popović, Martina Šašić; lifeStage: Adult; **Taxon:** kingdom: Animalia; phylum: Arthropoda; class: Insecta; order: Lepidoptera; family: Nymphalidae; genus: Maniola; specificEpithet: jurtina; scientificNameAuthorship: (Linnaeus, 1758); **Location:** continent: Europe; country: Hungary; decimalLatitude: 46.180634; decimalLongitude: 19.897794; geodeticDatum: WGS84; **Identification:** identifiedBy: Miloš Popović, Martina Šašić; **Event:** samplingProtocol: Observation; eventDate: 2015/08/06; year: 2015; month: 8; day: 6; **Record Level:** type: Dataset; modified: WedWed/FebFeb/20162016; language: English; rightsHolder: Miloš Popović
- **Type status:** Other material. **Occurrence:** recordedBy: Miloš Popović, Martina Šašić; lifeStage: Adult; **Taxon:** kingdom: Animalia; phylum: Arthropoda; class: Insecta; order: Lepidoptera; family: Lycaenidae; genus: Phengaris; specificEpithet: teleius; scientificNameAuthorship: (Bergsträsser, 1779); **Location:** continent: Europe; country: Hungary; decimalLatitude: 46.180634; decimalLongitude: 19.897794; geodeticDatum: WGS84; **Identification:** identifiedBy: Miloš Popović, Martina Šašić; **Event:** samplingProtocol: Observation; eventDate: 2015/08/06; year: 2015; month: 8; day: 6; **Record Level:** type: Dataset; modified: WedWed/FebFeb/20162016; language: English; rightsHolder: Miloš Popović
- **Type status:** Other material. **Occurrence:** recordedBy: Miloš Popović, Martina Šašić; lifeStage: Adult; **Taxon:** kingdom: Animalia; phylum: Arthropoda; class: Insecta; order: Lepidoptera; family: Lycaenidae; genus: Polyommatus; specificEpithet: icarus; scientificNameAuthorship: (Rottemburg, 1775); **Location:** continent: Europe; country: Hungary; decimalLatitude: 46.180634; decimalLongitude: 19.897794; geodeticDatum: WGS84; **Identification:** identifiedBy: Miloš Popović, Martina Šašić; **Event:** samplingProtocol: Observation; eventDate: 2015/08/06; year: 2015; month: 8; day: 6; **Record Level:** type: Dataset; modified: WedWed/FebFeb/20162016; language: English; rightsHolder: Miloš Popović
- **Type status:** Other material. **Occurrence:** recordedBy: Miloš Popović, Martina Šašić; lifeStage: Adult; **Taxon:** kingdom: Animalia; phylum: Arthropoda; class: Insecta; order: Lepidoptera; family: Hesperiidae; genus: Hesperia; specificEpithet: comma; scientificNameAuthorship: (Linnaeus, 1758); **Location:** continent: Europe; country: Hungary; decimalLatitude: 46.180634; decimalLongitude: 19.897794; geodeticDatum: WGS84; **Identification:** identifiedBy: Miloš Popović, Martina Šašić; **Event:** samplingProtocol: Observation; eventDate: 2015/08/06; year: 2015; month: 8; day: 6; **Record Level:** type: Dataset; modified: WedWed/FebFeb/20162016; language: English; rightsHolder: Miloš Popović
- **Type status:** Other material. **Occurrence:** recordedBy: Miloš Popović, Martina Šašić; lifeStage: Adult; **Taxon:** kingdom: Animalia; phylum: Arthropoda; class: Insecta; order: Lepidoptera; family: Pieridae; genus: Leptidea; specificEpithet: sinapis; scientificNameAuthorship: (Linnaeus, 1758); **Location:** continent: Europe; country: Hungary; decimalLatitude: 46.180634; decimalLongitude: 19.897794; geodeticDatum: WGS84; **Identification:** identifiedBy: Miloš Popović, Martina Šašić; **Event:** samplingProtocol: Observation; eventDate: 2015/08/06; year: 2015; month: 8; day: 6; **Record Level:** type: Dataset; modified: WedWed/FebFeb/20162016; language: English; rightsHolder: Miloš Popović
- **Type status:** Other material. **Occurrence:** recordedBy: Miloš Popović, Martina Šašić; lifeStage: Adult; **Taxon:** kingdom: Animalia; phylum: Arthropoda; class: Insecta; order: Lepidoptera; family: Nymphalidae; genus: Melitaea; specificEpithet: phoebe; scientificNameAuthorship: (Denis & Schiffermüller, 1775); **Location:** continent: Europe; country: Hungary; decimalLatitude: 46.180634; decimalLongitude: 19.897794; geodeticDatum: WGS84; **Identification:** identifiedBy: Miloš Popović, Martina Šašić; **Event:** samplingProtocol: Observation; eventDate: 2015/08/06; year: 2015; month: 8; day: 6; **Record Level:** type: Dataset; modified: WedWed/FebFeb/20162016; language: English; rightsHolder: Miloš Popović
- **Type status:** Other material. **Occurrence:** recordedBy: Miloš Popović, Martina Šašić; lifeStage: Adult; **Taxon:** kingdom: Animalia; phylum: Arthropoda; class: Insecta; order: Lepidoptera; family: Papilionidae; genus: Papilio; specificEpithet: machaon; scientificNameAuthorship: Linnaeus, 1758; **Location:** continent: Europe; country: Hungary; decimalLatitude: 46.180634; decimalLongitude: 19.897794; geodeticDatum: WGS84; **Identification:** identifiedBy: Miloš Popović, Martina Šašić; **Event:** samplingProtocol: Observation; eventDate: 2015/08/06; year: 2015; month: 8; day: 6; **Record Level:** type: Dataset; modified: WedWed/FebFeb/20162016; language: English; rightsHolder: Miloš Popović
- **Type status:** Other material. **Occurrence:** recordedBy: Miloš Popović, Martina Šašić; lifeStage: Adult; **Taxon:** kingdom: Animalia; phylum: Arthropoda; class: Insecta; order: Lepidoptera; family: Lycaenidae; genus: Phengaris; specificEpithet: teleius; scientificNameAuthorship: (Bergsträsser, 1779); **Location:** continent: Europe; country: Hungary; decimalLatitude: 46.299895; decimalLongitude: 19.900399; geodeticDatum: WGS84; **Identification:** identifiedBy: Miloš Popović, Martina Šašić; **Event:** samplingProtocol: Observation; eventDate: 2015/08/06; year: 2015; month: 8; day: 6; **Record Level:** type: Dataset; modified: WedWed/FebFeb/20162016; language: English; rightsHolder: Miloš Popović
- **Type status:** Other material. **Occurrence:** recordedBy: Miloš Popović, Martina Šašić; lifeStage: Adult; **Taxon:** kingdom: Animalia; phylum: Arthropoda; class: Insecta; order: Lepidoptera; family: Lycaenidae; genus: Polyommatus; specificEpithet: icarus; scientificNameAuthorship: (Rottemburg, 1775); **Location:** continent: Europe; country: Hungary; decimalLatitude: 46.299895; decimalLongitude: 19.900399; geodeticDatum: WGS84; **Identification:** identifiedBy: Miloš Popović, Martina Šašić; **Event:** samplingProtocol: Observation; eventDate: 2015/08/06; year: 2015; month: 8; day: 6; **Record Level:** type: Dataset; modified: WedWed/FebFeb/20162016; language: English; rightsHolder: Miloš Popović
- **Type status:** Other material. **Occurrence:** recordedBy: Miloš Popović, Martina Šašić; lifeStage: Adult; **Taxon:** kingdom: Animalia; phylum: Arthropoda; class: Insecta; order: Lepidoptera; family: Nymphalidae; genus: Maniola; specificEpithet: jurtina; scientificNameAuthorship: (Linnaeus, 1758); **Location:** continent: Europe; country: Hungary; decimalLatitude: 46.299895; decimalLongitude: 19.900399; geodeticDatum: WGS84; **Identification:** identifiedBy: Miloš Popović, Martina Šašić; **Event:** samplingProtocol: Observation; eventDate: 2015/08/06; year: 2015; month: 8; day: 6; **Record Level:** type: Dataset; modified: WedWed/FebFeb/20162016; language: English; rightsHolder: Miloš Popović
- **Type status:** Other material. **Occurrence:** recordedBy: Miloš Popović, Martina Šašić; lifeStage: Adult; **Taxon:** kingdom: Animalia; phylum: Arthropoda; class: Insecta; order: Lepidoptera; family: Hesperiidae; genus: Erynnis; specificEpithet: tages; scientificNameAuthorship: (Linnaeus, 1758); **Location:** continent: Europe; country: Hungary; decimalLatitude: 46.299895; decimalLongitude: 19.900399; geodeticDatum: WGS84; **Identification:** identifiedBy: Miloš Popović, Martina Šašić; **Event:** samplingProtocol: Observation; eventDate: 2015/08/06; year: 2015; month: 8; day: 6; **Record Level:** type: Dataset; modified: WedWed/FebFeb/20162016; language: English; rightsHolder: Miloš Popović
- **Type status:** Other material. **Occurrence:** recordedBy: Miloš Popović, Martina Šašić; lifeStage: Adult; **Taxon:** kingdom: Animalia; phylum: Arthropoda; class: Insecta; order: Lepidoptera; family: Pieridae; genus: Leptidea; specificEpithet: sinapis; scientificNameAuthorship: (Linnaeus, 1758); **Location:** continent: Europe; country: Hungary; decimalLatitude: 46.299895; decimalLongitude: 19.900399; geodeticDatum: WGS84; **Identification:** identifiedBy: Miloš Popović, Martina Šašić; **Event:** samplingProtocol: Observation; eventDate: 2015/08/06; year: 2015; month: 8; day: 6; **Record Level:** type: Dataset; modified: WedWed/FebFeb/20162016; language: English; rightsHolder: Miloš Popović

#### Distribution

**Figure 1.**
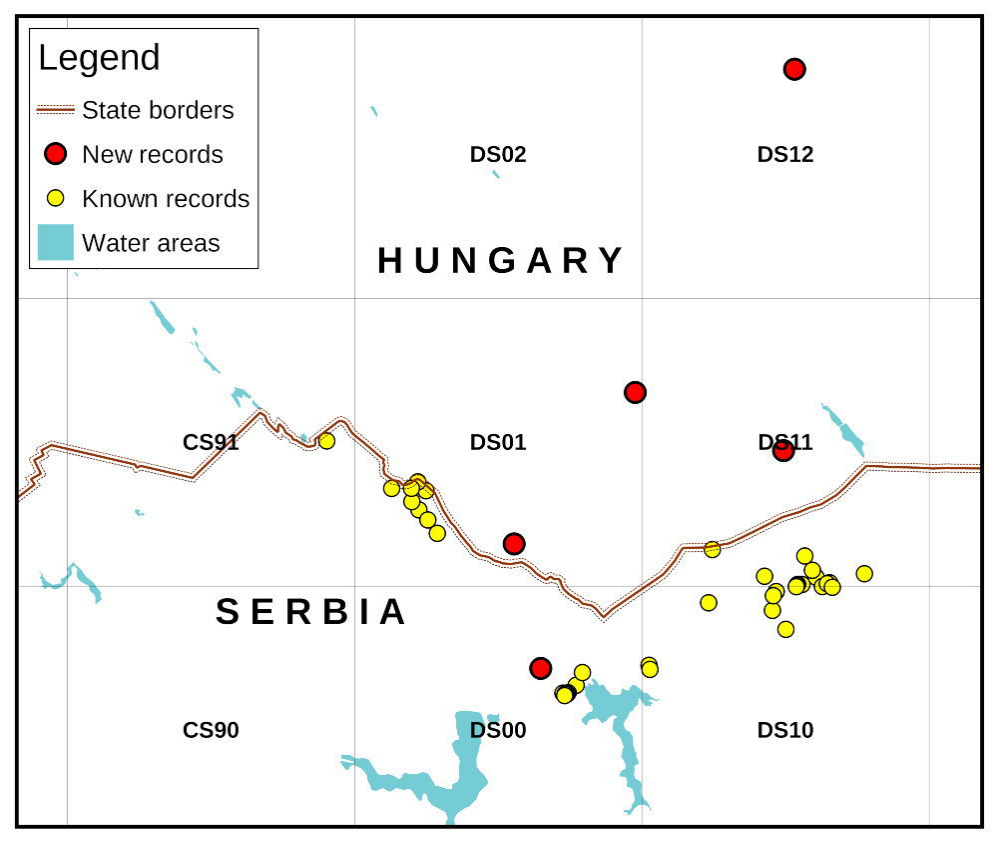
Distribution of *Phengaris teleius* in the bordering region between Serbia and Hungary shown on an UTM 10×10 km map (Zone 34T). Names of UTM fields are written in the centre of each square.

**Figure 2.**
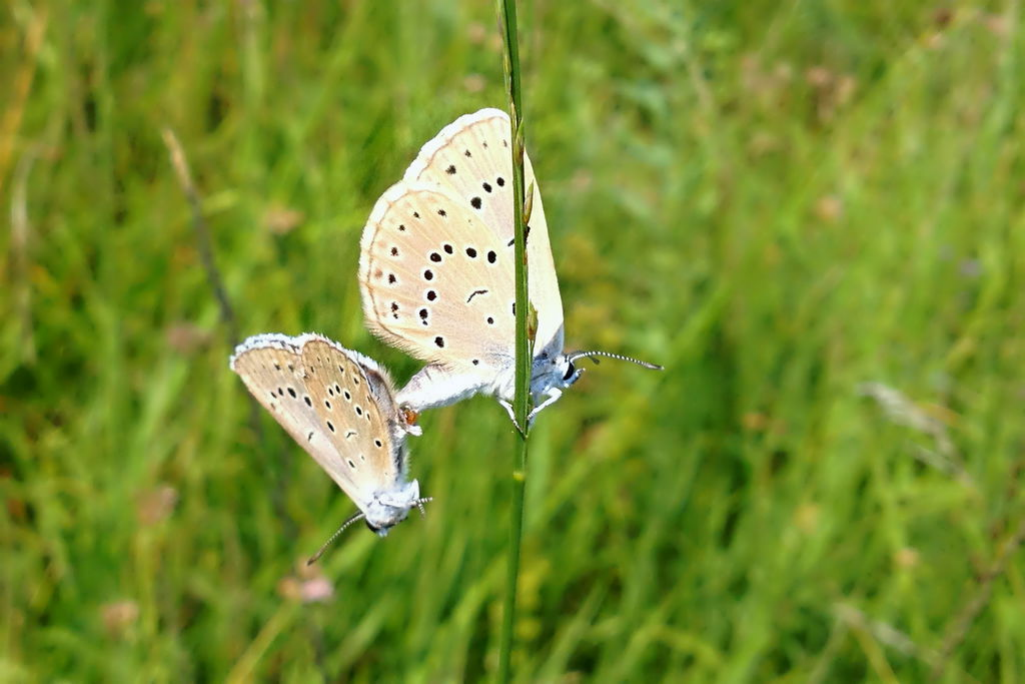
First observation of *Phengaris teleius* from south Hungary, photographed on wet meadows at Csipak semlyék. Photo: Martina Šašić, 6^th^ August 2015.

**Figure 3.**
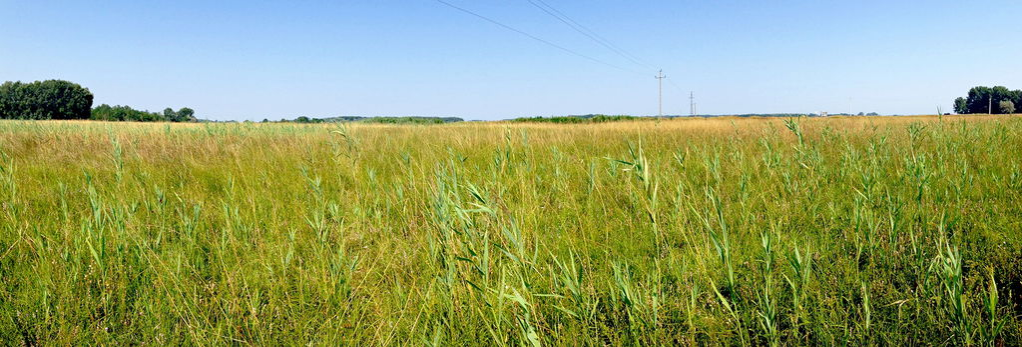
*Phengaris teleius* habitat in south Hungary near Ásotthalmi láprét (Csodarét). Photo: Martina Šašić, 6th August 2015.

**Figure 4.**
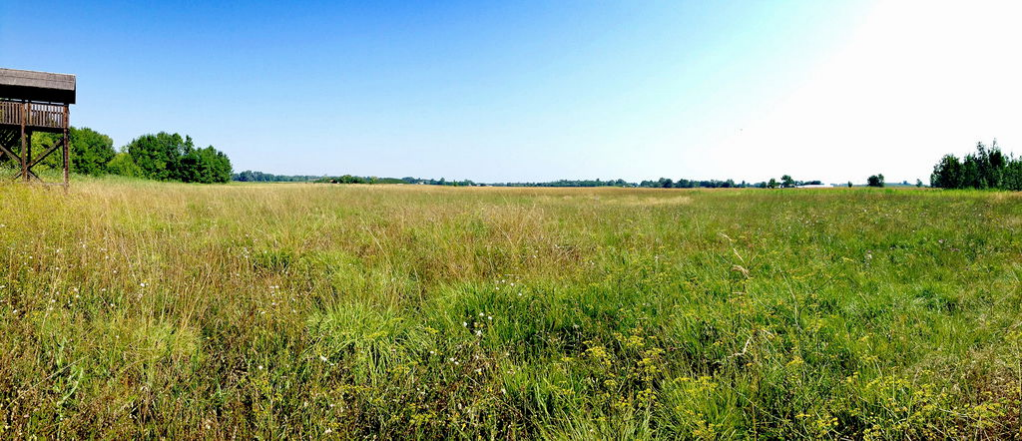
*Phegaris teleius* habitat in south Hungary at Csipak semlyék. Photo: Martina Šašić, 6th August 2015.

During this survey, *Phengaris teleius* was recorded in all visited localities in Hungary (Figs 1, 2). They are situated inside the National Park Kiskunság and are mown once a year to help sustaining populations of protected plants and animals (Figs 3, 4). In Serbia, the single unpublished observation is located on a motorbike polygon, close to the known populations at Ludaš lake (Fig. 1). The Serbian populations are also located inside nationally protected areas and are managed in similar manner to the Hungarian ones.

## Discussion

The new records of *Phengaris teleius* in Hungary and Serbia shows that the local populations may be more interconnected than previously thought (Popović et al. 2014). This could have great conservation importance, knowing the small migration distances and sedentary character of this butterfly species (Nowicki et al. 2005). As maximal migrations reach up to 5 km, at least some exchange of individuals is possible between local populations (Nowicki et al. 2014, Nowicki et al. 2005).

The distribution of *P. teleius* in Serbia is probably limited only to the study area and is now well known due to the considerable research efforts made since its discovery in 2012. However, more detailed studies are required in south-eastern Hungary for mapping its range and habitats. These populations are on the southern border of the species distribution in Europe (Wynhoff 1998), thus more conservation importance and efforts is required for their conservation (Bourn and Thomas 2002).

On some sites hosting *P. teleius*, we also observed the Large Copper, *Lycaena dispar* (Haworth 1802), another Natura 2000 species that can be found in wetland habitats. This increases the conservation value of the remaining wet meadow fragments. Such meadows are now among the most important habitats for the survival of threatened butterflies in Europe and the persistence of *P. teleius* depends on their appropriate management, including mowing regimes (Settele et al. 2005). In addition, the survival of this butterfly is highly dependent on the presence of host plant and host ants, both being sensitive to management practice. Recent study has shown that the local populations in Serbia are still exceptionally numerous, although a few local extinctions are recorded on a small scale (Popović, unpublished data). There is a need for additional research on ecology of *P. teleius* in order to adapt habitat management to the local needs of present butterfly populations. For example, in similar habitats in Hungary, low grazing was indicated as adequate management practice (Batáry et al. 2007) and mowing regimes have been proven important for its conservation in Serbia (Popović et al. 2014).

The results of scientific studies should be applied in joint conservation plans for *P. teleius* in both Serbia and Hungary, especially because the species conservation status is known to be unfavourable. This is easy to achieve since the studied populations are located inside of the protected areas, where some active management practice already exsist. The present locations of the species should be urgently included into the official ecological network of the Republic of Serbia and planned for inclusion within the upcoming Natura 2000 areas. Special attention should be given to preserve current water regimes inside the protected areas, create better connection between isolated habitats and monitor remaining populations of *P. teleius*.

## Supplementary Material

XML Treatment for Phengaris teleius

## References

[B2153475] Batáry Péter, Orvossy N., Korosi A., Nagy MARIANNA VÁLYI, Peregovits László (2007). Microhabitat preferences of *Maculinea
teleius* (Lepidoptera: Lycaenidae) in a mosaic landscape. European Journal of Entomology.

[B3036926] Bourn Nigel A. D, Thomas J. A (2002). The challenge of conserving grassland insects at the margins of their range in Europe. Biological Conservation.

[B2207117] Haraszty L. (2014). Natura 2000 fajok és élőhelyek Magyarországon.

[B3036950] Nowicki Piotr, Witek Madgalena, Skórka Piotr, Settele Josef, Woyciechowski Michal (2005). Population ecology of the endangered butterflies *Maculinea
teleius* and *Maculinea
nausithous* and the implications for conservation. Population Ecology.

[B3037506] Nowicki Piotr, Vrabec Vladimir, Binzenhöfer Birgit, Feil Johann, Zakšek Barbara, Hovestadt Thomas, Settele Josef (2014). Butterfly dispersal in inhospitable matrix: rare, risky, but long-distance. Landscape Ecology.

[B2153360] Popović Miloš, Radaković Miloš, Đurđević Aca, Franeta Filip, Verovnik Rudi (2014). Distribution and threats of *Phengaris
teleius* (Lepidoptera: Lycaenidae) in Northern Serbia. Acta Zoologica Academiae Scientiarum Hungaricae.

[B2153288] Settele Josef, Thomas Jeremy, Kühn Elisabeth (2005). Species ecology along a European gradient: *Maculinea* butterflies as a model.

[B2153345] Van Swaay Chris, Wynhoff Irma, Verovnik Rudi, Wiemers Martin, Munguira Miguel López, Maes Dirk, Šašić Martina, Verstrael Theo, Warren Martin, Settele Josef Phengaris
teleius. The IUCN Red List of Threatened Species. http://www.iucnredlist.org/details/12664/1.

[B2153335] Wynhoff Irma (1998). REVIEW: The recent distribution of the European *Maculinea* species. Journal of Insect Conservation.

